# Intravesical BCG-induced granulomatous prostatitis mimicking prostate cancer in patients with non-muscle invasive bladder cancer: a systematic review

**DOI:** 10.1007/s00345-026-06883-3

**Published:** 2026-11-05

**Authors:** Keiichiro Miyajima, Marcin Miszczyk, Maximilian J. Rabil, Felix Lübbersmeyer, Kazuma Tsuboi, Akihiro Matsukawa, Fumihiko Urabe, Keiichiro Mori, Takafumi Yanagisawa, Michael S. Leapman, Takahiro Kimura, Shahrokh F. Shariat

**Affiliations:** 1 Department of Urology, Comprehensive Cancer Center, Medical University of Vienna, Vienna, Austria; 2 Department of Urology, The Jikei University School of Medicine, Tokyo, Japan; 3 Collegium Medicum - Faculty of Medicine, WSB University, Dąbrowa Górnicza, Poland; 4 Department of Urology, Yale School of Medicine, New Haven, Connecticut USA; 5 Department of Urology, Medical University Center Hamburg-Eppendorf, Hamburg, Germany; 6 Department of Urology, Dentistry and Pharmaceutical Sciences, Okayama University Graduate School of Medicine, Okayama, Japan; 7 Department of Urology, University of Texas Southwestern Medical Center, Dallas, TX USA; 8 Department of Urology, Weill Cornell Medical College, New York, NY USA; 9 Hourani Center for Applied Scientific Research, Al-Ahliyya Amman University, Amman, Jordan; 10 College of Medical Health Technologies, Al-Turath University, Mansour, Baghdad Iraq; 11 Karl Landsteiner Institute of Urology and Andrology, Vienna, Austria; 12 Institute for Urology and Reproductive Health, I.M. Sechenov First Moscow State Medical University, Moscow, Russia; 13 Department of Urology, Comprehensive Cancer Center, Medical University of Vienna, Vienna General Hospital, Währinger Gürtel 18-20, Vienna, 1090 Austria

**Keywords:** Granulomatous prostatitis, Bacillus Calmette–Guérin, Non–muscle-invasive bladder cancer, Multiparametric magnetic resonance imaging, Prostate biopsy

## Abstract

**Purpose:**

Bacillus Calmette–Guérin (BCG) therapy is a standard-of-care for non–muscle-invasive bladder cancer (NMIBC), and granulomatous prostatitis (GP) is a side effect often misdiagnosed as prostate cancer (PCa). We conducted a systematic review to clarify the clinical spectrum of BCG-induced GP.

**Methods:**

MEDLINE, Embase, and Web of Science were searched in June 2026. We sought studies reporting GP associated with BCG therapy for NMIBC, including case reports and case series. Risk of bias was evaluated using ROBINS-I and JBI tools. Findings were synthesized narratively (CRD420261324758).

**Results:**

Forty-seven studies were included, comprising 23 case reports, 19 case series, and 5 comparative studies. In case series, abnormal digital rectal examination findings were reported in 100/240 (42%) evaluable cases. PSA elevation (> 4 ng/mL) occurred in 104/194 (54%) patients but often declined spontaneously during follow-up. Suspicious MRI findings were common, with PI-RADS ≥ 4 lesions reported in 71/82 (87%) cases, whereas biopsy-confirmed PCa was detected only in 12/151 (7.9%) patients. However, Grade Group and definitions of clinically significant PCa (csPCa) were incompletely and inconsistently reported, precluding a reliable estimate of csPCa frequency. Limitations include the predominance of descriptive studies and potential selection bias.

**Conclusion:**

BCG-induced GP frequently presents with transient PSA elevation and suspicious MRI findings that rarely indicate true PCa. In BCG-exposed patients with elevated PSA or PI-RADS ≥ 4 lesions, structured observation with careful follow-up should be considered before proceeding to immediate biopsy.

**Supplementary Information:**

The online version contains supplementary material available at https://doi.org/10.1007/s00345-026-06883-3.

## Introduction

Intravesical Bacillus Calmette–Guérin (BCG) therapy is a standard-of-care immune treatment for non–muscle-invasive bladder cancer (NMIBC) [1–3]. While highly effective, it is associated with several treatment-related adverse events, including the so-called granulomatous prostatitis (GP) [4]. The proposed pathophysiology involves intraprostatic reflux of BCG-containing urine through the prostatic ducts, leading to localized granulomatous inflammatory reactions. The BCG-induced GP remains incompletely characterized in the contemporary multiparametric magnetic resonance imaging (mpMRI) era. However, growing clinical observations suggest that it can closely mimic prostate cancer (PCa) via prostate-specific antigen (PSA) elevation and morphologic changes on mpMRI. Consequently, standard PCa diagnostic pathways, including PSA-based assessment and MRI-targeted biopsy strategies, may demonstrate reduced specificity in BCG-exposed patients, potentially leading to unnecessary prostate biopsy (PBx), patient anxiety, financial toxicity [5], and potential overtreatment in those with incidental findings of clinically-insignificant cancer.

Despite these important clinical implications, no comprehensive review has systematically evaluated its clinical presentation and imaging characteristics. In the absence of clear follow-up strategies or standardized diagnostic criteria, clinicians face uncertainty in balancing oncologic vigilance with avoidance of invasive procedures. Therefore, we conducted a systematic review to clarify the clinical spectrum of BCG-induced GP and to provide clinically relevant insights that may help recalibrate PCa workup strategies in BCG-exposed patients while maintaining oncologic safety.

## Methods

The protocol has been registered in the International Prospective Register of Systematic Reviews database (PROSPERO: CRD420261324758).

### Search strategy

This systematic review was reported in accordance with the Preferred Reporting Items for Systematic Reviews and Meta-Analyses (Supplementary Tables 1 and 2) [6] and adhered to the AMSTAR-2 checklist (Supplementary Appendix 1) [7]. On 24 February 2026, comprehensive literature searches were performed in MEDLINE (via PubMed), Embase, and the Web of Science Core Collection to identify studies reporting on GP associated with intravesical BCG therapy for NMIBC. An updated search was conducted on 1 June 2026. The complete search strategy is detailed in Supplementary Appendix 2.

The primary objective of this review was to clarify the clinical presentation, imaging characteristics, and histopathological features, and to provide clinically relevant insights to guide appropriate diagnostic evaluation and management. Two independent reviewers screened titles and abstracts to identify potentially eligible studies, followed by a full-text review of relevant articles. Any discrepancies were resolved through discussion and consensus with additional co-authors.

### Inclusion and exclusion criteria

We defined the study question using the PECO framework: In adult patients diagnosed with NMIBC (Population), does exposure to BCG therapy (Exposure), compared with patients not treated with BCG where applicable (Comparator), lead to clinical findings that may mimic PCa, including PSA elevation, abnormal digital rectal examination (DRE) findings, imaging abnormalities suggestive of prostate malignancy, and PCa detection rates on PBx (Outcomes). Because the primary aim of this review was descriptive, both comparative and non-comparative studies, including case reports and case series, were considered eligible. Studies without prostate-related evaluation or outcomes were excluded. Articles that did not provide original patient-level data, including review articles, editorials, letters without clinical information, and author replies, were excluded. Conference abstracts without full-text publications were excluded due to insufficient methodological and clinical detail.

### Data extraction

Data extraction was performed independently by two reviewers using a standardized data collection form. For each study, the following information was collected when available: first author’s name, year of publication, country, study design, number of patients, imaging modality, and diagnostic method. Potential cohort overlap was assessed by comparing study institutions, recruitment periods, study populations, and author groups across the included studies.

At the patient level, demographic and treatment-related variables included age, PSA levels before and after BCG therapy, DRE findings, imaging characteristics (including PI-RADS score), and PBx results. For case reports, management approaches (e.g., observation, antibiotics, anti-tuberculous therapy, biopsy, or surgery) and the subsequent clinical course were also documented. Any discrepancies in data extraction were resolved through discussion and consensus between the reviewers.

### Risk of bias assessment

Risk of bias (RoB) was assessed using the ROBINS-I tool for non-randomized comparative studies when applicable [8]. Case reports and case series were assessed using JBI Critical Appraisal Checklist [9]. Two authors independently assessed the RoB for each study. Any disagreements were resolved through discussion and consensus with co-authors.

### Data synthesis

Given the substantial clinical and methodological heterogeneity and the predominance of case reports and small case series, findings were synthesized narratively, and available data were summarized using descriptive statistics. Effect measures were not predefined. Reporting bias and certainty of evidence were not formally assessed due to the descriptive nature of data synthesis.

## Results

### Study selection and characteristics

**Fig. 1:**
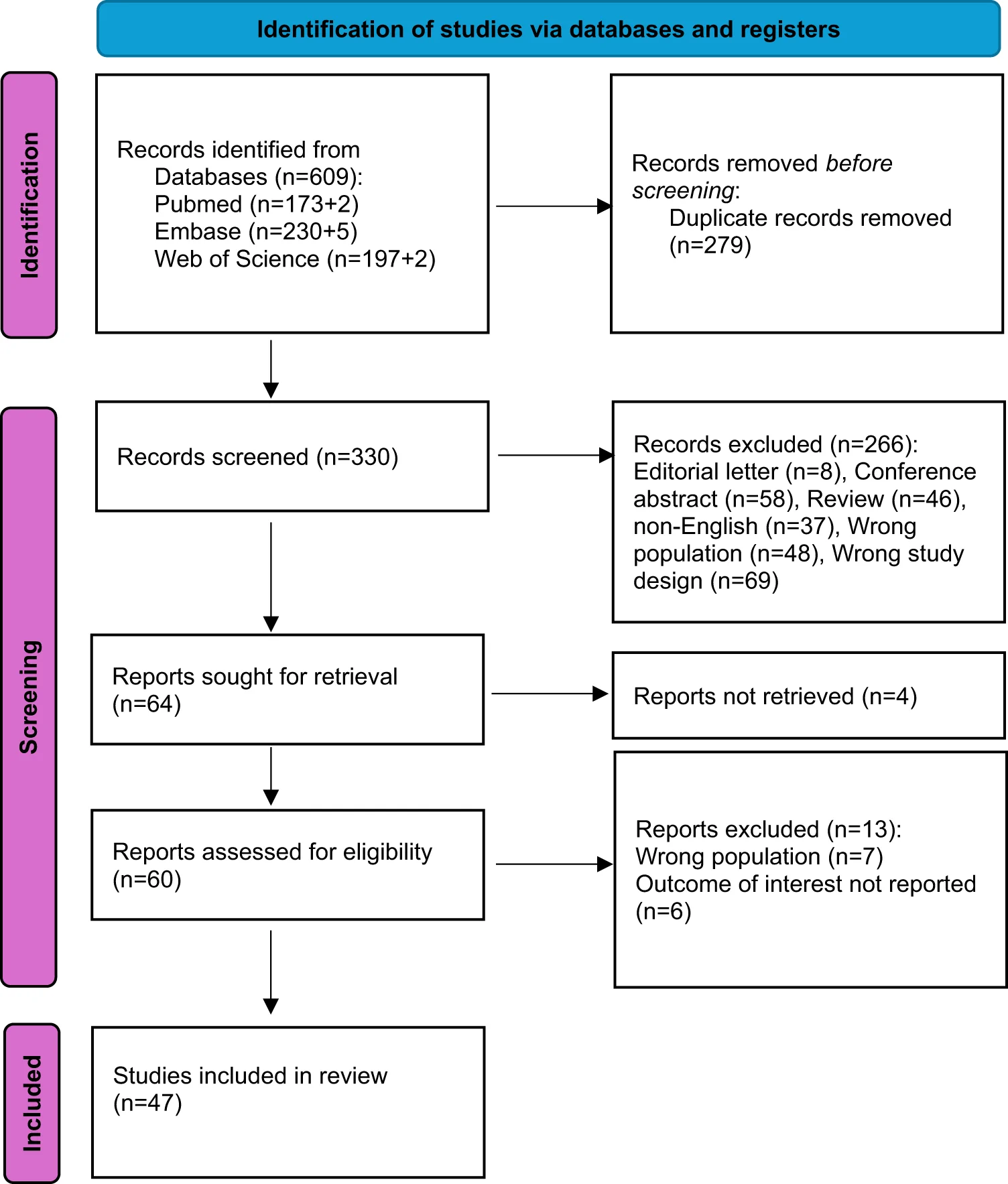
PRISMA 2020 flow diagram of study selection for the systematic review. Source: Page MJ, et al. BMJ 2021;372:n71. doi: https://doi.org/10.1136/bmj.n71. This work is licensed under CC BY 4.0. To view a copy of this license, visit https://creativecommons.org/licenses/by/4.0/

The study selection process is illustrated in Fig. 1. Forty-seven studies were included, comprising 23 case reports [10–32], 19 case series [33–51], and 5 non-randomized comparative studies [52–56]. Four studies were prospective [42, 45, 46, 53], and the remaining studies were retrospective. The overall characteristics of the included studies are summarized in Table 1. Detailed patient characteristics for case reports are shown in Supplementary Table 3.

#### Assessment of risk of bias and quality of study

The RoB assessment for each included comparative study is summarized in Supplementary Table 4. Based on the ROBINS-I tool, 3 studies were classified as having a moderate RoB, and 2 as having a serious RoB [52, 54]. The primary source of bias was selection bias, as several studies included only patients undergoing MRI or PBx after BCG therapy, which may not represent the overall BCG-treated population.

The methodological quality assessment of included case reports and case series based on the JBI critical appraisal tools is summarized in Supplementary Tables 5 and 6. Overall, most studies were considered to be of moderate to high quality, with histopathological confirmation and detailed imaging findings generally well reported. However, unclear consecutive patient inclusion and limited longitudinal follow-up were common limitations.

### Systematic review

Details of the included case series are presented in Tables 2 and 3, and Supplementary Table 7, while details of the included comparative studies are presented in Table 4. Because not all studies reported all clinical, imaging, pathological, and follow-up variables, the denominators vary across analyses and are reported separately for each outcome where applicable.

#### Clinical presentation

Across all included studies, the age of patients with BCG-induced GP ranged from 46 to 88 years, while the median age in studies reporting summary age data ranged from 64 to 74 years. Abnormal DRE findings were reported in 100 of 240 evaluable cases (42%) [35, 39, 42, 44, 46–51]. The indication for DRE varied across studies, from predefined follow-up to evaluation for suspected PCa. Notably, 22 of 36 patients (61%) in the case series with available data were asymptomatic at presentation [33, 39, 43, 48]. Case reports similarly described asymptomatic patients with suspicious DRE findings, often leading to biopsy for diagnostic confirmation.

In the comparative studies, Cabello et al. compared patients undergoing prostate MRI and targeted biopsy for suspected PCa with (*n* = 20) and without (*n* = 692) prior BCG exposure. Suspicious DRE findings were more frequent in the BCG-exposed group (30% vs. 19%), although the difference was not statistically significant (exact p-value not reported) [53]. Similarly, Akagi et al. compared BCG-exposed NMIBC patients undergoing PBx with non-BCG-exposed patients undergoing PBx for suspected PCa, and observed a higher proportion of abnormal DRE findings in the BCG-exposed group (*n* = 22) than in the non- exposed group (*n* = 28) (45% vs. 39%, *p* = 0.06) [52].

#### PSA findings

Among case series reporting PSA data, PSA elevation (≥ 4 ng/mL) was observed in 104 of 194 evaluable patients (54%) [33, 35–37, 39, 40, 43, 44, 46, 47, 51]. Reported PSA levels ranged from 0.6 to 22.9 ng/mL. PSA-related findings were reported in 3 case series [45–47]. Importantly, all case series that assessed PSA levels before and after BCG reported a spontaneous decline in PSA levels without specific treatment. PSA levels generally decreased toward baseline within approximately 3 months, although normalization took up to 6–12 months in some patients, suggesting that BCG-associated PSA elevation is transient and self-limiting (Table 2). Nonetheless, case reports described PSA elevation as a common trigger for further diagnostic evaluation and PBx.

Two comparative studies evaluated PSA levels according to prior BCG exposure. Neither Akagi et al. (5.8 vs. 6.3 ng/mL) nor Cabello et al. (median 6.6 vs. 6.4 ng/mL) found a significant difference between BCG-exposed and non-exposed patients [52, 53].

#### MRI findings

MRI-related findings were reported in 10 case series [35–44].MRI was generally performed for suspected PCa following PSA elevation and/or abnormal DRE, although some studies obtained MRI for bladder cancer staging or follow-up.

Among case series with reported PI-RADS assessment, PI-RADS ≥ 4 lesions were observed in 71 of 82 cases (87%) [35–42]. Four of these studies reported that MRI alone is insufficient to reliably distinguish GP from PCa [35, 36, 39, 44], while the remaining studies described specific imaging patterns that may help suggest GP. Typical MRI features included diffuse involvement, T1 hyperintensity, and ring enhancement patterns. Most lesions were nevertheless categorized as PI-RADS ≥ 4 (Table 3). Case reports similarly described lesions with suspicious mpMRI findings frequently classified as PI-RADS ≥ 4, often prompting PBx for diagnostic evaluation.

In Cabello et al., 19 of 20 BCG-exposed patients had at least one PI-RADS ≥ 4 lesion. At the lesion level, PCa (ISUP Grade Group [GG] ≥ 1) was detected in 3/18 PI-RADS 4 lesions (16.7%, 95%CI 5.8–39.2%) and 2/9 PI-RADS 5 lesions (22.2%, 95%CI 6.3–54.7%), compared with 61.6% (95%CI 57.3–65.8%) and 90.7% (95%CI 85.0-94.4%), respectively, in non-BCG-exposed patients [53]. Another comparative analysis demonstrated that diffuse morphology and T1 hyperintensity were characteristic MRI features of GP, which may aid in differentiating inflammatory lesions from PCa in selected cases (*n* = 99) [55]. Kim et al. reported that higher BMI and larger prostate volume were associated with the development of BCG-induced granulomatous lesions on MRI after BCG therapy (*n* = 194) [56].

#### Findings from other imaging modalities

Details of the findings from other imaging modalities are presented in Supplementary Table 7. Computed tomography (CT)-related findings were reported in 2 case series [33, 34]. CT was performed as part of oncological follow-up for bladder cancer rather than being specifically prompted by prostate-related symptoms. BCG-related GP was reported to present as prostatic enlargement or low-attenuation lesions with contrast enhancement, occasionally mimicking primary or metastatic tumors. In 1 series, incidental prostate abnormalities were observed in 11 of 38 patients (29%) and resolved spontaneously during follow-up [34].

Ultrasound (US)-related findings were reported in 4 case series [48–51]. On US, hypoechoic lesions were frequently observed, but these findings were generally nonspecific and often indistinguishable from PCa. Despite suspicious imaging findings, no biopsy-confirmed PCa was detected in these studies.

#### Histopathological findings

Histopathological confirmation was available in most case series, as the majority of studies included biopsy-proven GP. Among biopsy-confirmed cases, PCa was detected in 12 of 151 patients (7.9%) [33, 35–44, 46, 47, 49–51]. Grade Group was reported for 10 of these cancers: 5 were GG1, 4 were GG2, and 1 was GG5. Information on clinically significant PCa (csPCa) was inconsistently reported, and study-specific definitions were available in only 2 studies. Therefore, the overall frequency of csPCa could not be reliably estimated (Supplementary Table 8).

One comparative study demonstrated that PCa in BCG-exposed patients was detected only when biopsy was performed more than 1 year after treatment, whereas biopsies performed within 1 year showed no malignancy [52]. Hong et al. reported that PCa detection after BCG therapy was associated with bladder tumour location, with lower detection rates in patients with tumours involving the bladder neck or trigone compared with other locations (4.5% vs. 35%, *p* < 0.001; *n* = 59) [54].

## Discussion

To our knowledge, this is the first systematic review to comprehensively evaluate the clinical and imaging characteristics of BCG-induced GP, with a particular focus on its potential to mimic PCa. Our findings highlight several key associations. First, PSA elevation (> 4 ng/mL) was observed in 54% of evaluable patients, typically during or shortly after BCG induction, and subsequently declined spontaneously, with normalization occurring within 3–12 months. Second, although certain MRI patterns may suggest GP, PI-RADS ≥ 4 lesions were commonly observed after BCG therapy, with a relatively low rate of PCa detection. Finally, GP and PCa show substantial overlap in both clinical presentation and imaging findings, and histopathological confirmation by biopsy may be required when a definitive diagnosis is necessary.

In the setting of BCG therapy, PSA and mpMRI appear to be less specific for PCa detection. PSA elevation was frequently observed but often declined spontaneously during follow-up. Notably, PSA levels may decline even during maintenance BCG therapy. In a study incorporating maintenance BCG, PSA remained elevated at 3 months but declined by 6 months and approached baseline by 12 months despite ongoing maintenance therapy [46]. However, available evidence is insufficient to define an optimal timing for PSA assessment relative to individual maintenance cycles or a specific interval for deferring PBx. Similarly, suspicious MRI findings were common after BCG therapy, with PI-RADS ≥ 4 lesions frequently reported despite relatively low PCa detection rates. The timing of evaluation may also be relevant. Akagi et al. reported that PCa was identified only in patients undergoing PBx more than 1 year after BCG treatment, whereas biopsies performed within 1 year revealed only granulomatous inflammatory lesions [52]. Together with the observed spontaneous resolution of PSA and MRI abnormalities in several studies, these findings suggest that PSA and MRI abnormalities after BCG therapy may reflect inflammatory changes rather than malignancy.

From a histopathological perspective, BCG-induced GP is characterized by epithelioid granuloma formation with dense inflammatory infiltrates and occasional caseous necrosis. Pathological studies have shown that such lesions may occur more frequently than clinically recognized after intravesical BCG therapy; for example, GP was identified in 75% of cystoprostatectomy specimens from patients previously treated with BCG [57]. However, the interval between BCG exposure and cystoprostatectomy was not reported, limiting interpretation of the duration of these histopathological changes. Furthermore, granulomatous inflammation tends to involve the peripheral zone and follow the prostatic ductal system, suggesting that intraprostatic reflux of BCG-containing urine may contribute to its pathogenesis [58]. These pathological features likely explain why the inflammatory lesions can demonstrate diffusion restriction and enhancement patterns on mpMRI that mimic PCa. Crucially, because this granulomatous inflammation preferentially involves the peripheral zone—the most common location of PCa—BCG-induced GP can generate findings that closely resemble malignancy, thereby substantially reducing the specificity of PI-RADS-based assessment in this patient population.

When interpreting these suspicious prostate findings, the broader clinical context of patients receiving intravesical BCG therapy must be prioritized. These patients typically present with NMIBC, are often of advanced age, and frequently harbour multiple comorbidities. In many instances, the patient’s overall prognosis is primarily dictated by the clinical course of their bladder cancer rather than the presence of concomitant PCa. Therefore, the clinical benefit of detecting incidental, often low-grade PCa must be rigorously balanced against the risks of overdiagnosis and the morbidity associated with invasive procedures. Clinical decision-making in this setting should prioritize the optimal management of the primary bladder malignancy, maintaining a high threshold for aggressive prostate interventions.

Based on the available evidence, a pragmatic approach incorporating the timing of BCG exposure and the evolution of prostate findings may help guide clinical management. Establishing a baseline PSA value before BCG treatment may facilitate subsequent interpretation. Following BCG therapy, transient PSA elevation and suspicious imaging findings should be interpreted in the context of their evolution during follow-up. In the absence of persistent or strongly suspicious findings, structured observation with serial PSA testing and/or follow-up mpMRI may be a reasonable initial approach. PBx should be considered when PSA elevation persists, imaging abnormalities progress or remain suspicious, or other clinical features raise concern for PCa.

This study has several limitations. First, the majority of the included evidence relies on case reports and small case series, which are inherently prone to publication and reporting biases; because studies with striking findings are preferentially published, the overall certainty of evidence remains low. Second, substantial heterogeneity in patient demographics, BCG protocols, and imaging parameters precluded a formal quantitative meta-analysis. Third, many studies focused selectively on patients who underwent PBx or mpMRI due to clinical suspicion, introducing verification bias and potentially overrepresenting suspicious imaging findings. Therefore, the reported PCa detection rates do not reflect the true prevalence of PCa among all BCG-treated patients. Furthermore, Grade Group was incompletely reported, and definitions of csPCa were rarely provided and were not uniform across studies, precluding a reliable estimate of the frequency of csPCa. Finally, the lack of long-term follow-up and prospective comparative data makes it difficult to ascertain the natural history of BCG-induced GP, highlighting the need for well-designed prospective studies to optimize future diagnostic strategies.

## Conclusions

BCG-induced GP is an underrecognized condition that can closely mimic PCa. PSA elevation and suspicious MRI findings after BCG therapy may reflect transient inflammatory changes, potentially reducing the specificity of standard PCa diagnostic markers in this population. Therefore, even in BCG-exposed patients presenting with elevated PSA or PI-RADS ≥ 4 lesions, a strategy of structured observation and careful clinical follow-up could be considered before proceeding to immediate PBx, provided there are no persistent or progressing suspicious findings during longitudinal assessment. Such approach may help avoid unnecessary invasive procedures while maintaining oncologic safety.

**Table 1:** Basic characteristics of the included studies

Author, Year, Country	Study design	Population	*N*	BCG setting	Imaging modality	Diagnostic method
Case reports						
Bahnson et al., 1991 USA	R	Histologically-confirmed BCG-induced GP	1	I	US	PBx
Bialek et al., 2016 Poland	R	Histologically-confirmed BCG-induced GP	1	I	US	PBx
Bilsen et al., 2018 Netherlands	R	Histologically-confirmed BCG-induced GP	1	I	CT	Cx
Cadiz et al., 2016 Chile	R	Histologically-confirmed BCG-induced GP	1	I	US	PBx
de Leon et al., 2018 NR	R	Histologically-confirmed BCG-induced GP	1	NR	MRI	PBx
Dianat et al., 2014 USA	R	Histologically-confirmed BCG-induced GP	1	NR	MRI	PBx
Doo et al., 2012 Korea	R	Histologically-confirmed BCG-induced GP	1	I	CT	PBx
Garrido-Abad et al., 2018 Spain	R	Histologically-confirmed BCG-induced GP	1	NR	MRI	PBx
Gillard et al., 2022 Belgium	R	Histologically-confirmed BCG-induced GP	1	NR	MRI	PBx
Gundogdu et al., 2024 Turkey	R	Histologically-confirmed BCG-induced GP	1	NR	MRI	Cx
Hegde et al., 2024 USA	R	Histologically-confirmed BCG-induced GP	1	NR	MRI	PBx
Joaquim et al., 2012 Portugal	R	Histologically-confirmed BCG-induced GP	1	I + M	NR	PBx
Kovacic et al., 2026 Slovakia	R	Histologically-confirmed BCG-induced GP	1	I	MRI	PBx
Lee et al., 2018 Korea	R	Histologically-confirmed BCG-induced GP	1	I + M	US	PBx
Logan et al., 2014 USA	R	Histologically-confirmed BCG-induced GP	1	I	MRI	PBx
Nguyen et al., 2017 USA	R	Histologically-confirmed BCG-induced GP	1	I + M	MRI	PBx
Okcelik et al., 2013 Turkey	R	Histologically-confirmed BCG-induced GP	1	I	US	PBx
Rifkin et al., 1998 USA	R	Histologically-confirmed BCG-induced GP	1	NR	US	PBx
Salif et al., 2024 Belgium	R	Histologically-confirmed BCG-induced GP	1	I	US	PBx
Sarkis et al., 2020 Lebanon	R	Histologically-confirmed BCG-induced GP	1	I	MRI	PBx
Sauvat et al., 2021 France	R	Histologically-confirmed BCG-induced GP	1	I	MRI	PBx
Shibutani et al., 2023 Japan	R	Histologically-confirmed BCG-induced GP	1	I + M	MRI	Cx
Yu et al., 2025 USA	R	Histologically-confirmed BCG-induced GP	1	I	MRI	PBx
Case series						
Avudaiappan et al., 2024 USA	R	BCG-treated patients undergoing prostate evaluation	10	I + M	MRI	PBx
Bertelli et al., 2022 Italy	R	Histologically-confirmed BCG-induced GP	4	NR	MRI	PBx
Durmaz et al., 2026 Turkey	R	Histologically-confirmed BCG-induced GP	11	NR	MRI	PBx
Gottlieb et al., 2017 USA	R	Histologically-confirmed BCG-induced GP	6	NR	MRI	PBx
Guerra et al., 2024 Portugal	R	Histologically-confirmed BCG-induced GP	12	NR	MRI	PBx
Kawada et al., 2015 Japan	R	Histologically-confirmed BCG-induced GP	5	NR	MRI	PBx, Cx
Lee et al., 2022 Korea	R	Histologically-confirmed BCG-induced GP	24	NR	MRI	PBx, Cx
Ogreden et al., 2024 Turkey	P	BCG-treated patients undergoing prostate evaluation	10	I	MRI	PBx
Suzuki et al., 2013 Japan	R	Histologically-confirmed BCG-induced GP	10	I + M	MRI	PBx
Wang et al., 2021 China	R	Histologically-confirmed BCG-induced GP	8	NR	MRI	PBx
Ahmadi et al., 2025 Iran	P	BCG-treated patients undergoing PSA follow-up	20	I	NR	NR
Beltrami et al., 2008 Italy	P	BCG-treated patients undergoing PSA/DRE follow-up	77	I + M	NR	PBx, Cx
Leibovici et al., 2000 Israel	R	BCG-treated patients undergoing PSA/DRE follow-up	36	I	NR	PBx, Cx
Ma et al., 2009 USA	R	Patients with BCG-related prostatic granulomatous involvement	8	NR	CT	PBx, Cx
Matsushima et al., 2017 Japan	R	BCG-treated patients undergoing CT follow-up	38	I	CT	NR
Miyashita et al., 1992 USA	R	Histologically-confirmed BCG-induced GP	6	I + M	US	Cx
Mukamel et al., 1990 Israel	R	BCG-treated patients undergoing DRE/TRUS follow-up	36	I + M	US	PBx
Oates et al., 1988 USA	R	BCG-treated patients undergoing prostate follow-up	32	I + M	US	PBx
Terris et al., 1997 USA	R	BCG-treated patients undergoing prostate evaluation	13	I + M	US	PBx, Cx
Comparative study						
Akagi et al., 2024 Japan	R	Patients undergoing PBx for suspected PCa (non-BCG vs. BCG)	28/22	NR	MRI	PBx
Cabello et al., 2024 Spain	P	Patients undergoing MRI/PBx for suspected PCa (non-BCG vs. BCG)	692/20	I + M	MRI	PBx
Hong et al., 2014 Korea	R	BCG-treated patients undergoing PBx (bladder neck/trigone vs. other tumor location)	22/37	I	MRI	PBx
Ito et al., 2026 Japan	R	Patients with PCa vs. BCG-induced GP	88/11	NR	MRI	PBx, Cx
Kim et al., 2020 Korea	R	BCG-treated patients evaluated for post-BCG prostate MRI changes (negative vs. GP)	144/50	I	MRI	NR

CT, computed tomography; Cx, cystoprostatectomy; GP, granulomatous prostatitis; I, induction; M, maintenance; MRI, magnetic resonance imaging; NR, not reported; P, prospective; PBx, prostate biopsy; R, retrospective; TURP, transurethral resection of the prostate; US, ultrasound

N represents the study population relevant to the outcomes included in this systematic review and does not necessarily represent the number of patients with histologically confirmed BCG-induced GP. For comparative studies, sample sizes are presented according to the comparison groups shown in the Study population column

**Table 2:** PSA findings in the included case series

Author, Year, Country	Age, years	Baseline PSA, ng/mL	Peak PSA after BCG, ng/mL	PSA at 3 months, ng/mL	Biopsy performed, *n* (%)	PCa detected, *n* (%)	Key findings
Ahmadi et al., 2025 Iran	64 ± 8	0.7 (IQR 0.2-2.0)	1.5 (IQR 0.9–3.2)	0.8 (IQR 0.3-2.0)	0/20 (0)	0/20 (0)	A strategy of “watchful waiting” with repeat PSA testing at three months may prevent unnecessary prostate biopsies, reduce patient anxiety, and optimize healthcare resource utilization.
Beltrami et al., 2008 Italy	71 ± 8	1.6 (IQR 0.8–2.4)	3.5 (IQR 1.5–5.5)	3.7 (IQR 1.6–5.4)	10/77 (14)	1/77 (1.4)	PSA elevation was transient, with normalization by 6–12 months; biopsy could be deferred with monitoring.
Leibovici et al., 2000 Israel	NR	1.3	3.8	NR	6/36 (21)	0/36 (0)	Intravesical BCG therapy is associated with significantly elevated PSA in up to 40% of cases. This effect is self-limited, and PSA reverts to normal in 3 months.

BCG, Bacillus Calmette–Guérin; PSA, prostate-specific antigen; PCa, prostate cancer; IQR, interquartile range; NR, not reported

**Table 3:** MRI findings in the included case series

Author, Year, Country	Age, years	PSA, ng/mL	PI-RADS > = 4, *n* (%)	Biopsy-confirmed PCa, *n* (%)	Key findings
Avudaiappan et al., 2024 USA	NR	6.8 (6.1–10.5)	8/10 (80)	3/10 (30)	High mpMRI sensitivity (100%) but low specificity (29%) for BCG-related GP.
Bertelli et al., 2022 Italy	62 (52–72)	8.0 (7-22.9)	4/4 (100)	0	Persistent high-peak enhancement is commonly observed in BCG-related GP.
Durmaz et al., 2026 Turkey	72 (54–76)	6.0 (3.6–11.2)	10/11 (91)	1/11 (9)	Hyperintensity on T1-WI may serve as a distinguishing feature for BCG-related GP.
Gottlieb et al., 2017 USA	69 (50–79)	NR	1/6 (17)	0	BCG-related GP showed two MRI patterns: a common PI-RADS 3 chronic phase that may be managed with surveillance and a less frequent PI-RADS ≥ 4 acute phase that may require biopsy.
Guerra et al., 2024 Portugal	63 (46–75)	7.3 (4.6–11.2)	12/12 (100)	0	GP is difficult to distinguish from clinically significant PCa using clinical assessment and imaging alone.
Kawada et al., 2015 Japan	70 (60–79)	3.2 (1.8–6.3)	5/5 (100)	0	Prolonged ring enhancement may suggest BCG-induced GP.
Lee et al., 2022 Korea	66 (50–88)	NR	22/24 (92)	5/20 (25)	Contrast-enhanced T1-weighted imaging and DWI may play a role in its differential diagnosis from PCa.
Ogreden et al., 2024 Turkey	67 (55–87)	4.3	9/10 (90)	1/10 (10)	BCG increases PSA value and causes abnormal findings of mpMRI.
Suzuki et al., 2013 Japan	70 (62–81)	7.4 (1.7–16.1)	NR	0	The three main patterns of GP identified were diffuse, nodular, and cystic with a mural nodule. The diffuse type was the most common.
Wang et al., 2021 China	67 (47–76)	6.8 (2.0-12.9)	NR	0	BCG-related lesions in the peripheral zone showed low T2WI signal, high DWI signal, and low ADC signal, mimicking prostate cancer.

BCG, Bacillus Calmette–Guérin; GP, granulomatous prostatitis; PI-RADS, Prostate Imaging Reporting and Data System; PCa, prostate cancer; PSA, prostate-specific antigen; mpMRI, multiparametric magnetic resonance imaging; NR, not reported

**Table 4:** Summary of included comparative studies

Author, Year, Country	Comparison (G1 vs. G2)	Median time from BCG to evaluation, months	*N*		Age, years		PSA, ng/mL		PI-RADS score > = 4, *n* (%)		PCa detection rate (%)		Key findings
			G1	G2	G1	G2	G1	G2	G1	G2	G1	G2	
MRI													
Cabello et al., 2024 Spain	non-BCG group vs. BCG group	3–36 (MRI)	692	20	68 (61–74)	72 (63–77)	6.4 (5–9)	6.6 (5-11.3)	561 (81)	19 (95)	57	17	PCa detection in PI-RADS ≥ 4 lesions was significantly lower in BCG-treated patients than in non-BCG patients.
Ito et al., 2026 Japan	PCa group vs. GP group	30 (9–74) (MRI)	88	11	69 (48–80)	69 (48–80)	49 (3.2–1506)	8.8 (4.3–23.1)	NR	NR	100	0	T1 hyperintensity and a diffuse morphology were characteristic features of GP on MRI.
Timing of biopsy													
Akagi et al., 2024 Japan	non-BCG group vs. BCG group	NR (PBx)	28	22	71 (54–89)	74 (58–86)	6.3 (2.1–20.8)	5.8 (1.2–26)	NR	NR	79	14	PCa in BCG-treated patients was detected only in biopsies performed > 1 year after therapy.
Clinical factors													
Hong et al., 2014 Korea	bladder neck and/or trigone group vs. other location group	17 ± 12 (PBx)	22	37	69 ± 8	68 ± 6	6.1 ± 3.6	5.1 ± 3.9	NR	NR	4.5	35	PCa detection after BCG was associated with bladder tumor location.
Kim et al., 2020 Korea	negative group vs. GP group	3.1 (0.6–11.2) (MRI)	144	50	67 ± 11	66 ± 11	2.5 ± 3.2	3.8 ± 3.4	NR	21 (42)	NR	NR	BMI and prostate volume predicted post-BCG prostate lesions on MRI.

BCG, Bacillus Calmette–Guérin; BMI, body mass index; GP, granulomatous prostatitis; G1, group 1; G2, group 2; MRI, magnetic resonance imaging; PCa, prostate cancer; PI-RADS, Prostate Imaging Reporting and Data System; PSA, prostate-specific antigen; NR, not reported

## Supplementary Information

Below is the link to the electronic supplementary material.

Supplementary Material 1

## Data Availability

No new datasets were generated or analysed in this study. All data are available from the cited published literature.

## Abbreviations

BCG: Bacillus Calmette–Guérin
csPCa: Clinically significant prostate cancer
CT: Computed tomography
DRE: Digital rectal examination
GP: Granulomatous prostatitis
mpMRI: Multiparametric magnetic resonance imaging
NMIBC: Non–muscle-invasive bladder cancer
PBx: Prostate biopsy
PCa: Prostate cancer
PI-RADS: Prostate imaging reporting and data system
PSA: Prostate-specific antigen
RoB: Risk of bias
ROBINS-I: Risk of bias in non-randomized studies of interventions
US: Ultrasound
