# Supplementary material for: Intravesical BCG-induced granulomatous prostatitis mimicking prostate cancer in patients with non-muscle invasive bladder cancer: a systematic review
Source: World J Urol. 2026 Nov 5;44(1):787. doi: 10.1007/s00345-026-06883-3 (PMC13740009; doi:10.1007/s00345-026-06883-3)
Supplement: Supplementary file 1 [file 345_2026_6883_MOESM1_ESM.docx]

**Supplementary Information**

**Supplementary Table 1.** PRISMA 2020 for Abstracts Checklist

**Supplementary Table 2.** PRISMA 2020

**Supplementary Table 3.** Detailed patient characteristics of the included case reports

**Supplementary Table 4.** Risk of bias assessment of included comparative studies (ROBINS-I)

**Supplementary Table 5.** Overview of JBI checklist for case reports

**Supplementary Table 6.** Overview of JBI checklist for case series

**Supplementary Table 7.** Findings from other imaging modalities

**Supplementary Table 8.** Prostate cancer detection and pathological characteristics among patients undergoing prostate biopsy in the included case series

**Supplementary Appendix 1**. AMSTAR-2 checklist

**Supplementary Appendix 2**. Search strategy for systematic review

**Supplementary Table 1.** PRISMA 2020 for Abstracts Checklist

| **Section and Topic** | **Item #** | **Checklist item** | **Reported (Yes/No)** |
| --- | --- | --- | --- |
| **TITLE** | | |  |
| Title | 1 | Identify the report as a systematic review. | Yes |
| **BACKGROUND** | | |  |
| Objectives | 2 | Provide an explicit statement of the main objective(s) or question(s) the review addresses. | Yes |
| **METHODS** | | |  |
| Eligibility criteria | 3 | Specify the inclusion and exclusion criteria for the review. | Yes |
| Information sources | 4 | Specify the information sources (e.g. databases, registers) used to identify studies and the date when each was last searched. | Yes |
| Risk of bias | 5 | Specify the methods used to assess risk of bias in the included studies. | Yes |
| Synthesis of results | 6 | Specify the methods used to present and synthesise results. | Yes |
| **RESULTS** | | |  |
| Included studies | 7 | Give the total number of included studies and participants and summarise relevant characteristics of studies. | Yes |
| Synthesis of results | 8 | Present results for main outcomes, preferably indicating the number of included studies and participants for each. If meta-analysis was done, report the summary estimate and confidence/credible interval. If comparing groups, indicate the direction of the effect (i.e. which group is favoured). | Yes |
| **DISCUSSION** | | |  |
| Limitations of evidence | 9 | Provide a brief summary of the limitations of the evidence included in the review (e.g. study risk of bias, inconsistency and imprecision). | Yes |
| Interpretation | 10 | Provide a general interpretation of the results and important implications. | Yes |
| **OTHER** | | |  |
| Funding | 11 | Specify the primary source of funding for the review. | No |
| Registration | 12 | Provide the register name and registration number. | Yes |

**Supplementary Table 2.** PRISMA 2020

| **Section and Topic** | **Item #** | **Checklist item** | **Location where item is reported** |
| --- | --- | --- | --- |
| **TITLE** | | |  |
| Title | 1 | Identify the report as a systematic review. | Title |
| **ABSTRACT** | | |  |
| Abstract | 2 | See the PRISMA 2020 for Abstracts checklist. | Supplementary Table 1 |
| **INTRODUCTION** | | |  |
| Rationale | 3 | Describe the rationale for the review in the context of existing knowledge. | Introduction |
| Objectives | 4 | Provide an explicit statement of the objective(s) or question(s) the review addresses. | Introduction |
| **METHODS** | | |  |
| Eligibility criteria | 5 | Specify the inclusion and exclusion criteria for the review and how studies were grouped for the syntheses. | Methods |
| Information sources | 6 | Specify all databases, registers, websites, organisations, reference lists and other sources searched or consulted to identify studies. Specify the date when each source was last searched or consulted. | Methods, Figure 1, Supplementary Appendix 2 |
| Search strategy | 7 | Present the full search strategies for all databases, registers and websites, including any filters and limits used. | Supplementary Appendix 2 |
| Selection process | 8 | Specify the methods used to decide whether a study met the inclusion criteria of the review, including how many reviewers screened each record and each report retrieved, whether they worked independently, and if applicable, details of automation tools used in the process. | Methods |
| Data collection process | 9 | Specify the methods used to collect data from reports, including how many reviewers collected data from each report, whether they worked independently, any processes for obtaining or confirming data from study investigators, and if applicable, details of automation tools used in the process. | Methods |
| Data items | 10a | List and define all outcomes for which data were sought. Specify whether all results that were compatible with each outcome domain in each study were sought (e.g. for all measures, time points, analyses), and if not, the methods used to decide which results to collect. | Methods |
|  | 10b | List and define all other variables for which data were sought (e.g. participant and intervention characteristics, funding sources). Describe any assumptions made about any missing or unclear information. | Methods |
| Study risk of bias assessment | 11 | Specify the methods used to assess risk of bias in the included studies, including details of the tool(s) used, how many reviewers assessed each study and whether they worked independently, and if applicable, details of automation tools used in the process. | Methods |
| Effect measures | 12 | Specify for each outcome the effect measure(s) (e.g. risk ratio, mean difference) used in the synthesis or presentation of results. | Methods |
| Synthesis methods | 13a | Describe the processes used to decide which studies were eligible for each synthesis (e.g. tabulating the study intervention characteristics and comparing against the planned groups for each synthesis (item #5)). | Methods |
|  | 13b | Describe any methods required to prepare the data for presentation or synthesis, such as handling of missing summary statistics, or data conversions. | Methods |
|  | 13c | Describe any methods used to tabulate or visually display results of individual studies and syntheses. | Methods |
|  | 13d | Describe any methods used to synthesize results and provide a rationale for the choice(s). If meta-analysis was performed, describe the model(s), method(s) to identify the presence and extent of statistical heterogeneity, and software package(s) used. | Methods |
|  | 13e | Describe any methods used to explore possible causes of heterogeneity among study results (e.g. subgroup analysis, meta-regression). | Methods |
|  | 13f | Describe any sensitivity analyses conducted to assess robustness of the synthesized results. | Methods |
| Reporting bias assessment | 14 | Describe any methods used to assess risk of bias due to missing results in a synthesis (arising from reporting biases). | Methods |
| Certainty assessment | 15 | Describe any methods used to assess certainty (or confidence) in the body of evidence for an outcome. | Methods |
| **RESULTS** | | |  |
| Study selection | 16a | Describe the results of the search and selection process, from the number of records identified in the search to the number of studies included in the review, ideally using a flow diagram. | Results, Figure 1 |
|  | 16b | Cite studies that might appear to meet the inclusion criteria, but which were excluded, and explain why they were excluded. | Figure 1 |
| Study characteristics | 17 | Cite each included study and present its characteristics. | Results, Table 1 |
| Risk of bias in studies | 18 | Present assessments of risk of bias for each included study. | Supplementary Table 4 |
| Results of individual studies | 19 | For all outcomes, present, for each study: (a) summary statistics for each group (where appropriate) and (b) an effect estimate and its precision (e.g. confidence/credible interval), ideally using structured tables or plots. | Results, Table 2-4, Supplementary Table 5 |
| Results of syntheses | 20a | For each synthesis, briefly summarise the characteristics and risk of bias among contributing studies. | Results |
|  | 20b | Present results of all statistical syntheses conducted. If meta-analysis was done, present for each the summary estimate and its precision (e.g. confidence/credible interval) and measures of statistical heterogeneity. If comparing groups, describe the direction of the effect. | Results |
|  | 20c | Present results of all investigations of possible causes of heterogeneity among study results. | Results |
|  | 20d | Present results of all sensitivity analyses conducted to assess the robustness of the synthesized results. | Not applicable |
| Reporting biases | 21 | Present assessments of risk of bias due to missing results (arising from reporting biases) for each synthesis assessed. | Results |
| Certainty of evidence | 22 | Present assessments of certainty (or confidence) in the body of evidence for each outcome assessed. | Not applicable |
| **DISCUSSION** | | |  |
| Discussion | 23a | Provide a general interpretation of the results in the context of other evidence. | Discussion |
|  | 23b | Discuss any limitations of the evidence included in the review. | Discussion |
|  | 23c | Discuss any limitations of the review processes used. | Discussion |
|  | 23d | Discuss implications of the results for practice, policy, and future research. | Discussion |
| **OTHER INFORMATION** | | |  |
| Registration and protocol | 24a | Provide registration information for the review, including register name and registration number, or state that the review was not registered. | Methods |
|  | 24b | Indicate where the review protocol can be accessed, or state that a protocol was not prepared. | Methods |
|  | 24c | Describe and explain any amendments to information provided at registration or in the protocol. | Methods |
| Support | 25 | Describe sources of financial or non-financial support for the review, and the role of the funders or sponsors in the review. | Acknowledgements |
| Competing interests | 26 | Declare any competing interests of review authors. | Conflicts of interest |
| Availability of data, code and other materials | 27 | Report which of the following are publicly available and where they can be found: template data collection forms; data extracted from included studies; data used for all analyses; analytic code; any other materials used in the review. | All relevant data is presented in Tables, Figures, or described in the manuscript. Only simple analytic code was used for the study. The template data collection forms were not made public. |

**Supplementary Table 3.** Detailed patient characteristics of the included case reports

| Author, Year, Country | Age, years | pre-BCG PSA, ng/mL | Symptomatic (Yes/No) | PSA at diagnosis, ng/mL | Suspicious DRE at diagnosis (Y/N) | PI-RADS score | Treatment | PSA trend after diagnosis |
| --- | --- | --- | --- | --- | --- | --- | --- | --- |
| Bahnson et al., 1991 US | 67 | 4 | No | 6.9 | Yes | NR | NR | Not decreased |
| Bialek et al., 2016 Poland | 61 | 2.1 | Yes | NR | Yes | NR | Fluoroquinolone | Decreased |
| Bilsen et al., 2018 Netherlands | 66 | NR | Yes | 13.3 | NR | NR | Anti-TB therapy | NR |
| Cadiz et al., 2016 Chile | 75 | 2 | Yes | NR | NR | NR | Anti-TB therapy | NR |
| de Leon et al., 2018 NR | 68 | NR | No | 7.5 | NR | 3 | NR | NR |
| Dianat et al., 2014 US | 77 | <4 | No | 2.5 | Yes | 4 | NR | NR |
| Doo et al., 2012 Korea | 56 | 0.2 | Yes | 2.2 | Yes | NR | Anti-TB therapy | NR |
| Garrido-Abad et al., 2018 Spain | 63 | NR | No | 6.1 | NR | 4 | NR | NR |
| Gillard et al., 2022 Belgium | 66 | NR | No | 4.8 | Yes | 5 | NR | NR |
| Gundogdu et al., 2024 Turkey | 65 | 3.2 | No | 9.1 | Yes | 4 | Anti-TB therapy | Decreased |
| Hegde et al., 2024 US | 70 | NR | No | 6.4 | NR | 4 | NR | NR |
| Joaquim et al., 2012 Portugal | 58 | NR | No | 6 | NR | NR | Anti-TB therapy | Decreased |
| Kovacic et al., 2026 Slovakia | 71 | 1.9 | No | 10 | NR | 5 | Anti-TB therapy | Decreased |
| Lee et al., 2018 Korea | 59 | 3.1 | No | 4.1 | NR | NR | Anti-TB therapy | Decreased |
| Logan et al., 2014 US | 78 | 4.7 | No | 10.7 | NR | 3 | No treatment | Decreased |
| Nguyen et al., 2017 US | 68 | NR | No | 2.5 | Yes | 5 | NR | NR |
| Okcelik et al., 2013 Turkey | 70 | NR | Yes | 10.1 | Yes | NR | Anti-TB therapy | Decreased |
| Rifkin et al., 1998 US | 48 | NR | No | 1.3 | Yes | NR | No treatment | NR |
| Salif et al., 2024 Belgium | 65 | NR | Yes | NR | NR | NR | Anti-TB therapy | NR |
| Sarkis et al., 2020 Lebanon | 72 | 6.5 | No | 21.6 | No | 5 | No treatment | Decreased |
| Sauvat et al., 2021 France | 65 | 1.7 | No | 8.5 | Yes | NR | Anti-TB therapy | Decreased |
| Shibutani et al., 2023 Japan | 77 | NR | Yes | NR | No | 4 | Anti-TB therapy | NR |
| Yu et al., 2025 US | 64 | 3.6 | No | 10.3 | NR | 4 | No treatment | NR |
| Abbreviations: BCG, Bacillus Calmette–Guérin; DRE, digital rectal examination; NR, not reported; PI-RADS, Prostate Imaging Reporting and Data System; PSA, prostate-specific antigen; TB, tuberculosis. | | | | | | | | |

**Supplementary Table 4.** Risk of bias assessment of included comparative studies (ROBINS-I)

| Study | D1 | D2 | D3 | D4 | D5 | D6 | D7 | Overall |
| --- | --- | --- | --- | --- | --- | --- | --- | --- |
| Akagi et al., 2024 | Moderate | Serious | Low | Low | Low | Low | Moderate | Serious |
| Cabello et al., 2024 | Moderate | Moderate | Low | Low | Low | Low | Low | Moderate |
| Hong et al., 2014 | Moderate | Serious | Low | Low | Low | Low | Low | Serious |
| Ito et al., 2026 | Moderate | Moderate | Low | Low | Low | Low | Moderate | Moderate |
| Kim et al., 2020 | Moderate | Moderate | Low | Low | Low | Moderate | Low | Moderate |
| bias due to confounding (D1); bias in the selection of participants into the study (D2); bias in the classification of interventions (D3); bias due to deviations from intended interventions (D4); bias due to missing data (D5); bias in the measurement of outcomes (D6); and bias in the selection of the reported result (D7) | | | | | | | | |

**Supplementary Table 5.** Overview of JBI checklist for case reports

| Author | Q1 | Q2 | Q3 | Q4 | Q5 | Q6 | Q7 | Q8 | Overall appraisal |
| --- | --- | --- | --- | --- | --- | --- | --- | --- | --- |
| Bahnson et al., 1991 | Yes | Yes | Yes | Yes | Yes | Yes | Yes | Yes | Include |
| Bialek et al., 2016 | Yes | Yes | Yes | Yes | Yes | Yes | Yes | Yes | Include |
| Bilsen et al., 2018 | Yes | Yes | Yes | Yes | Yes | Yes | Yes | Yes | Include |
| Cadiz et al., 2016 | Yes | Yes | Yes | Yes | Yes | Yes | Yes | Yes | Include |
| de Leon et al., 2018 | Yes | Yes | Yes | Yes | Unclear | Unclear | Yes | Yes | Include |
| Dianat et al., 2014 | Yes | Yes | Yes | Yes | Yes | Yes | Yes | Yes | Include |
| Doo et al., 2012 | Yes | Yes | Yes | Yes | Yes | Yes | Yes | Yes | Include |
| Garrido-Abad et al., 2018 | Yes | Yes | Yes | Yes | Yes | Unclear | Yes | Yes | Include |
| Gillard et al., 2022 | Yes | Yes | Yes | Yes | Yes | Unclear | Yes | Yes | Include |
| Gundogdu et al., 2024 | Yes | Yes | Yes | Yes | Yes | Yes | Yes | Yes | Include |
| Hegde et al., 2024 | Yes | Yes | Yes | Yes | Yes | Yes | Yes | Yes | Include |
| Joaquim et al., 2012 | Yes | Yes | Yes | Yes | Yes | Yes | Yes | Yes | Include |
| Kovacic et al., 2026 | Yes | Yes | Yes | Yes | Yes | Yes | Yes | Yes | Include |
| Lee et al., 2018 | Yes | Yes | Yes | Yes | Yes | Yes | Yes | Yes | Include |
| Logan et al., 2014 | Yes | Yes | Yes | Yes | Yes | Yes | Yes | Yes | Include |
| Nguyen et al., 2017 | Yes | Yes | Yes | Yes | Yes | Unclear | Yes | Yes | Include |
| Okcelik et al., 2013 | Yes | Yes | Yes | Yes | Yes | Yes | Yes | Yes | Include |
| Rifkin et al., 1998 | Yes | Yes | Yes | Yes | Yes | Yes | Yes | Yes | Include |
| Salif et al., 2024 | Yes | Yes | Yes | Yes | Yes | Yes | Yes | Yes | Include |
| Sarkis et al., 2020 | Yes | Yes | Yes | Yes | Yes | Yes | Yes | Yes | Include |
| Sauvat et al., 2021 | Yes | Yes | Yes | Yes | Yes | Yes | Yes | Yes | Include |
| Shibutani et al., 2023 | Yes | Yes | Yes | Yes | Yes | Yes | Yes | Yes | Include |
| Yu et al., 2025 | Yes | Yes | Yes | Yes | Yes | Yes | Yes | Yes | Include |
| Q1: Were the patient’s demographic characteristics clearly described?  Q2: Was the patient’s history clearly described and presented as a timeline?  Q3: Was the current clinical condition of the patient on presentation clearly described?  Q4: Were diagnostic tests or assessment methods and the results clearly described?  Q5: Was the intervention(s) or treatment procedure(s) clearly described?  Q6: Was the post-intervention clinical condition clearly described?  Q7: Were adverse events (harms) or unanticipated events identified and described?  Q8: Does the case report provide takeaway lessons? | | | | | | | | | |

**Supplementary Table 6.** Overview of JBI checklist for case series

| Author | Q1 | Q2 | Q3 | Q4 | Q5 | Q6 | Q7 | Q8 | Q9 | Q10 | Overall appraisal |
| --- | --- | --- | --- | --- | --- | --- | --- | --- | --- | --- | --- |
| Avudaiappan et al., 2024 | Yes | Yes | Yes | Unclear | Uncliear | Yes | Yes | Yes | Yes | Yes | Included |
| Bertelli et al., 2022 | Yes | Yes | Yes | Yes | Yes | Yes | Yes | Yes | Yes | Yes | Included |
| Durmaz et al., 2026 | Yes | Yes | Yes | Unclear | Yes | Yes | Yes | No | Yes | Yes | Included |
| Gottlieb et al., 2017 | Yes | Yes | Yes | Unclear | Unclear | Yes | Yes | No | Yes | Yes | Included |
| Guerra et al., 2024 | Yes | Yes | Yes | Yes | Yes | Yes | Yes | Yes | Yes | Yes | Included |
| Kawada et al., 2015 | Yes | Yes | Yes | Unclear | Unclear | Yes | Yes | Yes | Yes | Yes | Included |
| Lee et al., 2022 | Yes | Yes | Yes | Yes | Yes | Yes | Yes | Yes | Yes | Yes | Included |
| Ogreden et al., 2024 | Yes | Yes | Yes | Unclear | Unclear | Yes | Yes | No | Yes | Yes | Included |
| Suzuki et al., 2013 | Yes | Yes | Yes | Unclear | Unclear | Yes | Yes | No | Yes | Yes | Included |
| Wang et al., 2021 | Yes | Yes | Yes | Unclear | Unclear | Yes | Yes | No | Yes | Yes | Included |
| Ahmadi et al., 2025 | Yes | Yes | Yes | Yes | No | Yes | Yes | Yes | No | Yes | Included |
| Beltrami et al., 2008 | Yes | Yes | Yes | Yes | No | Yes | Yes | Yes | Not applicalble | Yes | Included |
| Leibovici et al., 2000 | Yes | Yes | Yes | Yes | Yes | Yes | Yes | Yes | Yes | Yes | Included |
| Ma et al., 2009 | Yes | Yes | Yes | Unclear | No | Yes | Yes | Yes | Yes | Yes | Included |
| Matsushima et al., 2017 | Yes | Yes | Yes | Yes | Yes | Yes | Yes | Yes | Yes | Yes | Included |
| Miyashita et al., 1992 | Yes | Yes | Yes | Unclear | Unclear | Yes | Yes | No | Yes | Yes | Included |
| Mukamel et al., 1990 | Yes | Yes | Yes | Yes | Yes | Yes | Yes | Yes | Yes | Yes | Included |
| Oates et al., 1988 | Yes | Yes | Yes | Yes | Yes | Yes | Yes | Yes | Yes | Yes | Included |
| Terris et al., 1997 | Yes | Yes | Yes | Unclear | Yes | Yes | Yes | No | Yes | Yes | Included |
| Q1: Were there clear criteria for inclusion in the case series? Q2: Was the condition measured in a standard, reliable way for all participants included in the case series? Q3: Were valid methods used for identification of the condition for all participants included in the case series? Q4: Did the case series have consecutive inclusion of participants? Q5: Did the case series have complete inclusion of participants? Q6: Was there clear reporting of the demographics of the participants in the study? Q7: Was there clear reporting of clinical information of the participants? Q8: Were the outcomes or follow up results of cases clearly reported? Q9: Was there clear reporting of the presenting site(s)/clinic(s) demographic information? Q10: Was statistical analysis appropriate? | | | | | | | | | | | |

**Supplementary Table 7.** Findings from other imaging modalities

| Author, Year, Country | Age, years | PSA, ng/mL | Imaging findings, n (%) | Biopsy performed, n (%) | Biopsy-confirmed PCa, n (%) | Key findings |
| --- | --- | --- | --- | --- | --- | --- |
| **CT studies** |  |  |  |  |  |  |
| Ma et al., 2009 USA | 68 (45-81) | NR | Enlarged prostate: 7/8 (88) | 3/8 (38) | 0/8 (0) | BCG-related granulomatous disease may mimic primary or metastatic tumors on CT. |
| Matsushima et al., 2017 Japan | 70±10 | NR | Low attenuation area: 6/38 (16) Contrast enhancement: 3/38 (7.8) Both findings: 2/38 (5.2) | NR | NR | Incidental prostate abnormalities were observed in 29% of patients and resolved spontaneously during follow-up. |
| **US studies** |  |  |  |  |  |  |
| Miyashita et al., 1992 USA | NR | NR | Hypoechoic lesion: 6/6 (100) | NA | NA | US findings were indistinguishable from prostate cancer. |
| Mukamel et al., 1990 Israel | NR | NR | Hypoechoic lesion: 20/36 (56) | 20/36 (56) | 0/36 (0) | Hypoechoic lesions were common after BCG, but no cancer was detected. |
| Oates et al., 1988 USA | NR | NR | NR | 9/32 (28) | 0/32 (0) | Prostatic induration or PSA fluctuations after BCG are frequently due to GP and are often asymptomatic. |
| Terris et al., 1997 USA | 68 (54-83) | 4.9 (0.5-15) | Hypoechoic lesion: 9/13 (69) | 9/13 (69) | 1/13 (7.7) | Persistent PSA elevation may prompt biopsy despite nonspecific US findings. |
| Abbreviations: BCG, Bacillus Calmette–Guérin; CT, computed tomography; US, ultrasonography; PCa, prostate cancer; PSA, prostate-specific antigen; NR, not reported; NA, not applicable. | | | | | | |

**Supplementary Table 8.** Prostate cancer detection and pathological characteristics among patients undergoing prostate biopsy in the included case series

| Study | Patients undergoing PBx, n | PCa detected on PBx, | Grade Group of detected PCa | Study-defined csPCa, n | Study-defined csPCa definition |
| --- | --- | --- | --- | --- | --- |
| Avudaiappan et al. | 10 | 3 | GG1, n=1; GG2, n=2 | NR | NR |
| Bertelli et al. | 4 | 0 | NA | 0 | NR |
| Durmaz et al. | 11 | 1 | GG1, n=1 | 0 | ISUP GG ≥2 |
| Gottlieb et al. | 6 | 0 | NA | 0 | NR |
| Guerra et al. | 12 | 0 | NA | 0 | NR |
| Kawada et al. | 3 | 0 | NA | 0 | NR |
| Lee et al. | 20 | 5 | GG1, n=2; GG2, n=2, GG5, n=1 | 4 | Gleason score >7 on whole-prostate specimen, tumor volume >0.5 cc, or extraprostatic extension |
| Ogreden et al. | 10 | 1 | GG1, n=1 | NR | NR |
| Suzuki et al. | 10 | 0 | NA | 0 | NR |
| Wang et al. | 8 | 0 | NA | 0 | NR |
| Beltrami et al. | 10 | 1 | NR | NR | NR |
| Leibovici et al. | 6 | 0 | NA | 0 | NR |
| Ma et al. | 3 | 0 | NA | 0 | NR |
| Oates et al. | 9 | 0 | NA | 0 | NR |
| Mukamel et al. | 20 | 0 | NA | 0 | NR |
| Terris et al. | 9 | 1 | NR | NR | NR |
| Abbreviations: PBx, prostate biopsy; PCa, prostate cancer; csPCa, clinically significant prostate cancer; GG, Grade Group; NA, not applicable; NR, not reported. Study-defined csPCa was reported only when explicitly defined or classified as such in the original study; no uniform post hoc definition was applied. | | | | | |

**Supplementary Appendix 1**. AMSTAR-2 checklist

**Supplementary Appendix 2**. Search strategy for systematic review

MEDLINE (via PubMed) – original search: 24 February 2026; updated search: 1 June 2026

| #1 | "mycobacterium bovis"[MeSH Terms] OR "mycobacterium bovis"[Title/Abstract] OR "BCG"[Title/Abstract] OR "bacillus calmette guerin"[Title/Abstract] OR "bacille calmette guerin"[Title/Abstract] | 37499 + 434 |
| --- | --- | --- |
| #2 | "prostatitis"[MeSH Terms] OR "prostatitis"[Title/Abstract] | 8929 + 86 |
| #3 | "prost*"[Title/Abstract] AND ("granuloma*"[Title/Abstract] OR "inflammation"[Title/Abstract] OR "inflammatory"[Title/Abstract]) | 42514 + 569 |
| #4 | #1 AND (#2 OR #3) | 173 + 2 |

Embase – original search: 24 February 2026; updated search: 1 June 2026

| #1 | ‘mycobacterium bovis’:ti,ab OR BCG:ti,ab OR ‘bacillus calmette guerin’:ti,ab OR ‘bacille calmette guerin’:ti,ab | 42817 + 589 |
| --- | --- | --- |
| #2 | ‘prostatitis’:ti,ab | 10073 + 131 |
| #3 | prost*:ti,ab AND (granuloma*:ti,ab OR inflammation:ti,ab OR inflammatory:ti,ab) | 56998 + 886 |
| #4 | #1 and (#2 or #3)  (‘mycobacterium bovis’:ti,ab OR BCG:ti,ab OR ‘bacillus calmette guerin’:ti,ab OR ‘bacille calmette guerin’:ti,ab) and (‘prostatitis’:ti,ab or (prost*:ti,ab AND (granuloma*:ti,ab OR inflammation:ti,ab OR inflammatory:ti,ab))) | 230 + 5 |

Web of Science – original search: 24 February 2026; updated search: 1 June 2026

| #1 | #1 TS=Mycobacterium bovis  #2 TS=BCG  #3 TS=Bacillus Calmette-Guerin  #1 or #2 or #3 | 41644→40557 |
| --- | --- | --- |
| #2 | #1 TS=prostatitis | 6845→6401 |
| #3 | #1 TS=prost*  #2 TS=granuloma*  #3 TS=inflammation  #4 TS=inflammatory  #1 and (#2 or #3 or #4) | 55804→54013 |
| #4 | #1 and (#2 or #3) | 197→174 |
| Two additional records published after the original search date were identified during the updated search. | | |
